# Who is at risk? Core mental health symptoms and problematic cannabis use in Germany

**DOI:** 10.1186/s42238-026-00430-y

**Published:** 2026-06-05

**Authors:** Eva-Maria Krowartz, Larissa Schwarzkopf, Sally Olderbak, Stefanie J. Klug, Gabriele Koller, Esther Neumeier, Elena Gomes de Matos, Eva Hoch

**Affiliations:** 1 Department of Psychiatry and Psychotherapy, LMU University Hospital, LMU Munich, Munich, Germany; 2 IFT Institut Für Therapieforschung, Centre for Mental Health and Addiction Research, Munich, Germany; 3 Department Clinical Psychology and Psychotherapy, Charlotte Fresenius University, Munich, Germany; 4 Institute for Medical Information Processing, Biometry, and Epidemiology – IBE, LMU Munich, Munich, Germany; 5 Faculty for Applied Healthcare Sciences, Technical University of Deggendorf, Deggendorf, Germany; 6 TUM School of Medicine and Health, Chair of Epidemiology, Technical University of Munich, Munich, Germany

**Keywords:** Cannabis, Cannabis dependence, Cannabis use disorder, Problematic cannabis use, Mental health, Core symptom

## Abstract

**Background and Aims:**

Cannabis is one of the most used psychoactive substances worldwide. This study presents nationally representative data on core mental health symptoms and other characteristics among individuals in Germany who used cannabis in the past 12 months, stratified into those with and without problematic cannabis use.

**Methods:**

Data were drawn from the 10th wave (2021) of the Epidemiological Survey of Substance Abuse, a cross-sectional survey of adults aged 18–64 years living in private households in Germany. The analytic sample included 1,004 past-year cannabis users. Problematic cannabis use was assessed using the Severity of Dependence Scale with sex-specific cut-offs (≥ 2 for women, ≥ 4 for men). Core mental health symptoms were measured with the DIA-X-Core Screening Questionnaire and categorized into 0, 1–3, or ≥ 4 symptoms. Analyses were weighted to match the German population. Descriptive comparisons between individuals with and without problematic use employed χ^2^-tests. Multivariable logistic regression models examined associations between problematic use and cumulative symptom scores, adjusting for covariates. P-values were corrected using the Simes–Benjamini–Hochberg procedure.

**Findings:**

Among individuals with past-year cannabis use, 25.0% met criteria for problematic use. Compared with those without problematic use, these individuals were more likely to have a low educational level (27.2% vs. 10.8%), use cannabis weekly or almost daily (66.8% vs. 14.8%), begin use before age 15 (40.8% vs. 19.1%), currently use tobacco (73.1% vs. 49.7%), and report problematic amphetamine- (10.5% vs. 2.2%) and tobacco use (23.8% vs.14.0%). They were less likely to report no core mental health symptoms (12.7% vs. 25.3%) and more likely to report ≥ 4 symptoms (40.8% vs. 27.2%). Adjusted models showed that reporting ≥ 4 symptoms was positively associated with problematic use (aOR = 1.68; 95% CI [1.12–2.53]; *p*=.027), whereas having no symptoms was associated negatively (aOR = 0.41; 95% CI [0.23–0.73]; *p*=.006).

**Conclusion:**

Amid changing cannabis laws in Germany, this study identifies risk factors for problematic use and highlights that core mental health symptoms are associated with problematic cannabis use. The findings underscore the importance of early screening and targeted prevention strategies.

**Supplementary Information:**

The online version contains supplementary material available at 10.1186/s42238-026-00430-y.

## Introduction

Problematic cannabis use refers to continued consumption despite adverse consequences for social functioning, physical or mental health, or the well-being of others (Casajuana et al. 2016). The onset and progression of problematic use are shaped by a complex interaction of biological, psychological, and social factors that influence individual development from childhood through adolescence into adulthood (Connor et al. 2021). Problematic cannabis use represents a significant risk factor for the development of cannabis use disorder (CUD), particularly among individuals with frequent cannabis use and those with self-medication motives or co-occurring mental health conditions (Connor et al. 2021; Hasin 2020). Whereas CUD is a clinically defined diagnostic entity, problematic cannabis use refers to a broader pattern of consumption associated with adverse health or social consequences that may not meet diagnostic criteria (Connor et al. 2021). Among persons who consume cannabis, the risk of developing schizophrenic psychosis, affective disorders (e.g., depression or bipolar disorder) and anxiety disorders is elevated (Hoch and Preuss 2024). Problematic use and CUD were associated with higher prevalence rates of mental disorders (Agosti et al. 2002; Connor et al. 2021; Hoch and Preuss 2024; Lev-Ran et al. 2013; Regier et al. 1990; Schlossarek et al. 2016; Sorkhou et al. 2024), including mood disorders, anxiety disorders and personality disorders (Hasin et al. 2016). Mental disorders are characterized by core symptoms that are essential for diagnosis. Although these symptoms alone do not constitute a diagnosis, they represent the defining features required for clinical identification. In their absence, a diagnosis of the respective disorder is considered unlikely (Wittchen 1997).

Corresponding population-based evidence on the interplay between mental health issues and cannabis use patterns is important, given the widespread use of cannabis and its status as the most commonly used drug under international control (UNODC 2025). An estimated 8% of European adults (22.8 million, 15–64 years) used cannabis in 2024 (EMCDDA 2024). In Germany, 8.8% of adults aged 18 to 64 years - around 4.5 million people - reported cannabis use in 2021, with 2.4% (approximately 1.3 million) showing signs of problematic use (Rauschert et al. 2022). 12-month prevalence rose to 9.8% in 2024, including 260,000 individuals meeting the Diagnostic and Statistical Manual of Mental Disorders, Fourth Edition (DSM-4) criteria for abuse and 510,000 meeting the criteria for dependence (Olderbak et al. 2024). The legalization of recreational cannabis in high-income countries may contribute to increases in cannabis use, problematic use, and CUD, particularly among vulnerable groups with mental health predispositions who are more susceptible to developing problematic use patterns or CUD (Connor et al. 2021; Hall and Lynskey 2020). Identifying subgroups at risk to develop appropriate early intervention and care programs is particularly important, as recreational cannabis use was partially legalized in Germany in April 2024 (BMG 2024). The new legislation introduced a regulated framework allowing possession and limited private as well as collective cultivation within officially sanctioned cultivation associations. Evidence from the first interim evaluation report (EKOCAN) suggests that, during the first 18 months after the law entered into force, no substantial changes have been observed across many of the assessed domains related to cannabis use and its regulation (Manthey et al. 2025). The establishment of representative data prior to legalization is therefore critical to ensure robust future evaluations of the partial legalization, particularly with respect to potentially vulnerable subgroups.

Using data from a survey designed to be nationally representative of the German adult population, this study is the first to compare individuals with and without problematic cannabis use with respect to core mental health symptoms, sociodemographic characteristics, consumption patterns, and the problematic use of other substances, thereby providing baseline data for subsequent analyses and identifying risk factors.

## Methods

### Study design and sampling

Data stem from the 2021 wave of the German Epidemiological Survey on Substance Abuse (ESA), a cross-sectional survey conducted on a triennial basis. The target population comprised German-speaking adults aged 18–64 years (birth cohorts 1957–2003) residing in private households in Germany, corresponding to approximately 51.1 million individuals (Statistisches Bundesamt 2020). The data collection was carried out from May to September 2021. A two-stage random sampling design was employed, with municipalities first randomly drawn based on municipal statistical data from the German Federal Statistical Office and the statistical offices of the German federal states, followed by random sampling of addresses from the corresponding population registers. To increase response rates and minimize sample selection bias, a mixed-methods approach was employed including paper-and-pencil questionnaires, computer-assisted-web interviews, and computer-assisted-telephone interviews. All participants provided informed consent and agreed to the processing of their data for the purposes of the research described. Further details on the methodology are published elsewhere (Rauschert et al. 2022).

### Instruments

#### Problematic cannabis use

Problematic use of cannabis was assessed using the Severity of Dependence Scale (SDS) (Gossop et al. 1995), which indicates problematic use of the respective substance over the past 12 months. The SDS has shown good reliability and internal consistency across substances (Gossop et al. 1995; Swift et al. 2012; van der Pol et al. 2013a)and has been validated for use in German population-based studies (Steiner and Kraus 2010). It consists of five items with the response options “never or almost never,” “sometimes,” “often,” and “always.” The total score ranges from 0 to 15, with different thresholds applied for different substances (Gossop et al. 1995; 2021). Participants were classified as having problematic cannabis use when their SDS score exceeded the sex-specific threshold (≥ 2 for women and ≥ 4 for men), while those below these thresholds were classified as having non-problematic use (Steiner and Kraus 2010).

#### Core mental health symptoms

The presence of core mental health symptoms was assessed using the DIA-X-Core Screening Questionnaire (DIA-X-SSQ), an established screening instrument developed by Wittchen and colleagues (Wittchen 1997), covering predefined core symptoms of agoraphobia, depression (dysthymia), generalized anxiety disorder, mania, panic disorder, post-traumatic stress disorder, social anxiety disorder, and somatoform disorder (Wittchen 1997). The DIA-X-SSQ is derived from the World Health Organization’s Composite International Diagnostic Interview (Robins et al. 1988) and assesses the presence of core symptoms of specific mental disorders over the past 12 months. For instance, a two-week period of sadness, low mood, and loss of interest is the core symptom of depression (Wittchen 1997). One item per core symptom was administered in the questionnaire, except for depression, which included two items. Original and translated items are provided in Supplementary material 1, with the translation of the original items conducted using *DeepL* (DeepL SE, Cologne, Germany). A cumulative symptom score (range: 0–8) was calculated by summing endorsed core symptoms to capture overall symptom burden across diagnostic domains. Given the high prevalence of comorbidity and the overlap in symptomatology across mental disorders, this approach reflects transdiagnostic vulnerability rather than isolated symptom presence. For analysis, the score was categorized into three groups (0, 1–3, ≥ 4) to enhance interpretability and to account for potential non-linear associations between symptom burden and problematic cannabis use. The categorization was informed by the empirical distribution of the data and ensured adequate cell counts for stable estimation.

#### Covariates of problematic cannabis use

The following covariates, which have been associated with problematic cannabis use, were also considered: sex (Cuttler et al. 2016; Khan et al. 2013); age (18–24 years, 25–34 years, 35–44 years, 45–54 years, 55 + years) (Hasin et al. 2016; Haug et al. 2017; Rubin-Kahana et al. 2022); education level (classified according to the International Standard Classification of Education, (ISCED) and grouped into three categories: low education (ISCED levels 0–2), middle education (ISCED levels 3–4), and high education (ISCED levels 5–8)) (Fergusson et al. 2003; Lynskey and Hall 2000; Schneider 2013); risk of poverty (below 60% of the median net equivalized income (Schlossarek et al. 2016; WSI 2022); cannabis use frequency (less than once a month, at least once a month, at least weekly, (almost) daily) (Hoch and Preuss 2024; Robinson et al. 2022); early age of cannabis initiation (≤ 15 years) (Le Strat et al. 2015; Millar et al. 2021); current tobacco use (Agrawal and Lynskey 2009; Badiani et al. 2015; Weinberger et al. 2020) (defined as having smoked at least 1 cigarette, cigar, cigarillo, or pipe within the past 30 days and > 100 cigarettes over the lifetime).

Using the SDS, problematic cocaine use was defined as a score ≥ 3 (Kaye and Darke 2002), and problematic amphetamine use as ≥ 5 (Topp and Mattick 1997). Problematic alcohol use was defined as ≥ 8 points on the Alcohol Use Disorders Identification Test (AUDIT) (Babor et al. 2001). Problematic tobacco use was assessed using the Fagerström Test for Nicotine Dependence (FTND), applying a cut-off score of ≥ 4 (Heatherton et al. 1991).

### Statistical analysis

Data were analyzed using *Stata *version 15.1 (StataCorp, College Station, TX, USA). All analyses were weighted using post-stratification weights to align the sample with the German population by federal state, municipality size, sex, birth cohort, and education (for details see (Rauschert et al. 2022)). The analytic sample included all individuals reporting cannabis use in the past 12 months (n=1,004). Variable-specific analyses were performed using available-case analysis, resulting in slight variations in sample size due to item-level missingness. Core mental health symptoms, sociodemographic characteristics, consumption patterns, and problematic use of other substances were compared between individuals with and without problematic cannabis, based on *χ*^*2*^-tests. Variables that showed significant group differences, along with sex, age, and interview mode - since previous findings from the ESA indicated mode effects on consumption estimates (Rauschert et al. 2022) - were included in three multivariable logistic regression models. Problematic tobacco use was excluded from the adjusted models due to high multicollinearity with current tobacco use. Logistic regression models were estimated with non-problematic cannabis use (vs. problematic use) as the dependent variable. These models estimated adjusted odds ratios (aORs) with 95% confidence intervals (CIs) and p-values. For the independent variable, core mental health symptoms were categorized into three mutually exclusive groups (0 symptoms; 1–3 symptoms; ≥ 4 symptoms). In the multivariable analyses, each symptom group was entered separately as a binary predictor (e.g., ≥ 4 symptoms vs. all others), such that individuals in the respective category were compared with all remaining participants. All models were adjusted for variables that showed statistically significant group differences in the bivariate analyses. To assess potential effect modification, interaction terms between the categorical core mental health symptom variable and sex as well as age group were examined in fully adjusted survey-weighted logistic regression models. To investigate whether the set of predictors significantly explained a proportion of the variance in problematic cannabis use, the global significance of each of the models was evaluated using design-based Wald tests (F-tests) and the corresponding p-values. To control for multiple testing across all inferential analyses (*χ2*-tests and logistic regression models), p-values were adjusted using the Simes–Benjamini–Hochberg procedure to control the false discovery rate (Simes 1986). Statistical significance was evaluated at a threshold of α < 0.05.

## Results

### Sample description

In the 2021 ESA, data were collected from 9,046 individuals, yielding a response rate of 35.0%. Among individuals who used cannabis in the past-12-months ( n=1,004), 25.0% were classified as having problematic cannabis use (n=231). Table 1 presents the sociodemographic characteristics of persons who used cannabis in the past 12 months, along with p-values from χ^2^-tests comparing individuals with problematic and non-problematic use.

**Table 1:** Sociodemographic characteristics of past 12-months cannabis users in Germany (n=1,004) by problematic cannabis use status

	**Past 12-month use ** **(** **n=1,004)**		**Non-problematic **^**a**^ ** use (** **n=773)**		**Problematic use **^**a**^**(n=231)**		p*	p**
	*n*	%	*n*	%	*n*	%		
Sociodemographic characteristics								
Gender (Male)	523	61.3	422	63.8	101	53.8	.060	.097
Age group							.220	.291
18–24	494	31.1	374	30.4	120	33.4	.442	.557
25–34	291	31.8	221	29.8	70	37.8	.084	.128
35–44	133	23.2	106	24.7	27	18.7	.214	.291
45–54	49	9.5	43	10.8	6	5.7	.183	.266
55–64	37	4.4	29	4.3	8	4.4	.986	.986
Education ^b^							** <.001**	** <.001**
Low	121	14.9	79	10.8	42	27.2	** <.001**	** <.001**
Middle	539	50.8	408	51.7	131	48.0	.461	.557
High	344	34.3	286	37.4	58	24.9	**.018**	**.034**
At risk of poverty ^c^	220	22.3	165	19.7	55	30.1	.051	.087

n is unweighted, percents are weighted

^a^Problematic cannabis use defined by the Severity of Dependence Scale (SDS): ≥ 2 for women, ≥ 4 for men (19 undefined)

^b^Based on the International Standard Classification of Education (ISCED) (Cuttler et al. 2016)

^c^Risk of poverty: < 60% of the median net equivalized income (Rubin-Kahana et al. 2022)

^*^Weighted chi-square tests were employed to test for differences between individuals with problematic and non-problematic use

^**^Adjusted using the Simes-Benjamini–Hochberg procedure to control the false discovery rate (Kaye and Darke 2002); significance threshold set at α < 0.05 and indicated with boldface

Significant differences between participants identified as having problematic and non-problematic patterns of use were observed in education, cannabis use frequency, early age of initiation, current tobacco use, problematic amphetamine use, and core mental health symptoms. Overall, 61.3% of all participants who used cannabis in the past 12 months were male, and the majority were aged between 18 and 34 years, with 31.1% aged 18–24 and 31.8% aged 25–34. Smaller proportions were aged 35–44 (23.2%), 45–54 (9.5%), or 55 and older (4.4%). Half of all respondents who reported cannabis use in the past 12 months (50.8%) reported a middle level of education, 34.3% had a high level, and 14.9% a low level of education. 22.3% were classified as being at risk of poverty. Low educational attainment was more prevalent among participants with problematic cannabis use (27.2% vs. 10.8%; *p* < 0.001), whereas high educational attainment was less prevalent in this group (24.9% vs. 37.4%; *p*=0.034) compared to those with non-problematic use. Consumption patterns, problematic use of other substances and current tobacco use, are shown in Table 2.

**Table 2:** Core mental health symptoms, cannabis use, and other problematic substances by problematic cannabis use status

	**Past 12-month use**			**Non-problematic use **^**a**^			**Problematic use **^**a**^			p*	p**
	* n (total)*	*n*	%	*n (total)*	*n*	%	*n (total)*	*n*	%		
Cannabis consumption patterns											
Frequency ^b^										** <.001**	** <.001**
Less than once a month	968	630	60.2	744	551	71.1	224	79	26.9	** <.001**	** <.001**
At least once a month	968	123	12.1	744	104	14.0	224	19	6.2	**.002**	**.006**
At least weekly	968	85	10.4	744	47	6.6	224	38	22.0	** <.001**	** <.001**
(almost) daily	968	130	17.3	744	42	8.2	224	88	44.8	** <.001**	** <.001**
Early age of initiation ^c^	1,004	237	24.5	773	148	19.1	231	89	40.8	** <.001**	** <.001**
Current tobacco use	1,001	474	55.6	771	322	49.7	230	152	73.1	** <.001**	** <.001**
Problematic use of other substances ^a^											
Alcohol	1,001	487	44.4	772	374	44.2	229	113	44.9	.876	.908
Amphetamine	979	27	4.1	760	13	2.2	219	14	10.5	**.001**	**.002**
Cocaine	990	27	3.4	766	19	3.0	224	8	4.4	.495	.574
Tobacco	1,004	106	16.4	773	64	14.0	231	42	23.8	**.027**	**.049**
Core mental health symptoms ^d^											
0	996	206	22.3	771	180	25.3	225	26	12.7	**.003**	**.007**
1–3	996	478	47.3	771	380	47.5	225	98	46.5	.843	.906
≥ 4	996	312	30.4	771	211	27.2	225	101	40.8	**.003**	**.006**

n is unweighted; percentages are weighted. All analyses are based on persons reporting cannabis use in the past 12 months ( n=1,004). Variation in total n across variables reflects item-specific missing data

^a^Problematic use defined by the Severity of Dependence Scale (SDS): cannabis ≥ 2 (women), ≥ 4 (men) (19 undefined); amphetamines ≥ 5 (Badiani et al. 2015); cocaine ≥ 3 (Millar et al. 2021). Problematic alcohol use: Alcohol Use Disorders Identification Test (AUDIT) ≥ 8 (Agrawal and Lynskey 2009). Problematic tobacco use: Fagerström Test for Nicotine Dependence (FTND) ≥ 4 (Weinberger et al. 2020)

^b^Self-reported frequency of cannabis use in the past 12 months

^c^Early age of cannabis initiation at ≤ 15 years

^d^Cumulative number of core mental health symptoms (range: 0–8), assessed using the DIA-X-SSQ (Wittchen 1997)

^*^Weighted chi-square tests were employed to test for differences between individuals with problematic and non-problematic use

^**^Adjusted using the Simes-Benjamini–Hochberg procedure to control the false discovery rate (Kaye and Darke 2002); significance threshold set at α < 0.05 and indicated with boldface

Most participants with use in the past-12-month reported consuming cannabis less than once a month (60.2%), while 17.3% used it almost daily. Individuals with non-problematic use were more likely to use cannabis infrequently, with 71.1% consuming less than once a month, compared to 26.9% among individuals with problematic use (*p* < 0.001). In contrast, frequent and intensive use were considerably more common among individuals with problematic use: 22.0% reported weekly use and 44.8% almost daily use, compared to 6.6% and 8.2% among participants with non-problematic use (both *p* < 0.001). An early onset of cannabis use (≤ 15 years) was reported by 24.5% of all participants who used cannabis in the past 12 months and was substantially more prevalent among those with problematic use (40.8%) than among those with non-problematic use (19.1%; *p*<0.001). More than half of participants who used cannabis smoked currently (55.6%), with a markedly higher proportion among participants with problematic use (73.1%) compared to those with non-problematic use (49.7%; *p* < 0.001). Regarding the use of other substances, problematic amphetamine use was significantly more common among participants with problematic cannabis use (10.5%) compared to those with non-problematic use (2.2%; *p*=0.002). In contrast, no significant group differences were found for problematic alcohol use among individuals with problematic use among those with non-problematic use (44.9% vs. 44.2%; *p*=0.911), problematic cocaine use (4.4% vs. 3.0%; *p*=0.560), or problematic tobacco use (23.8% vs. 14.0%; *p*=0.050).

Regarding core mental health symptoms, 22.3% of all individuals who reported cannabis use in the past 12 months reported no core mental health symptoms, 47.3% reported one to three symptoms, and 30.4% reported four or more symptoms. Individuals with problematic cannabis use were less likely to report no symptoms compared to those with non-problematic use (12.7% vs. 25.3%; *p*=0.007) and more likely to report four or more symptoms (40.8% vs. 27.2%; *p*=0.006).

### Problematic cannabis use and mental health

**Fig. 1:**
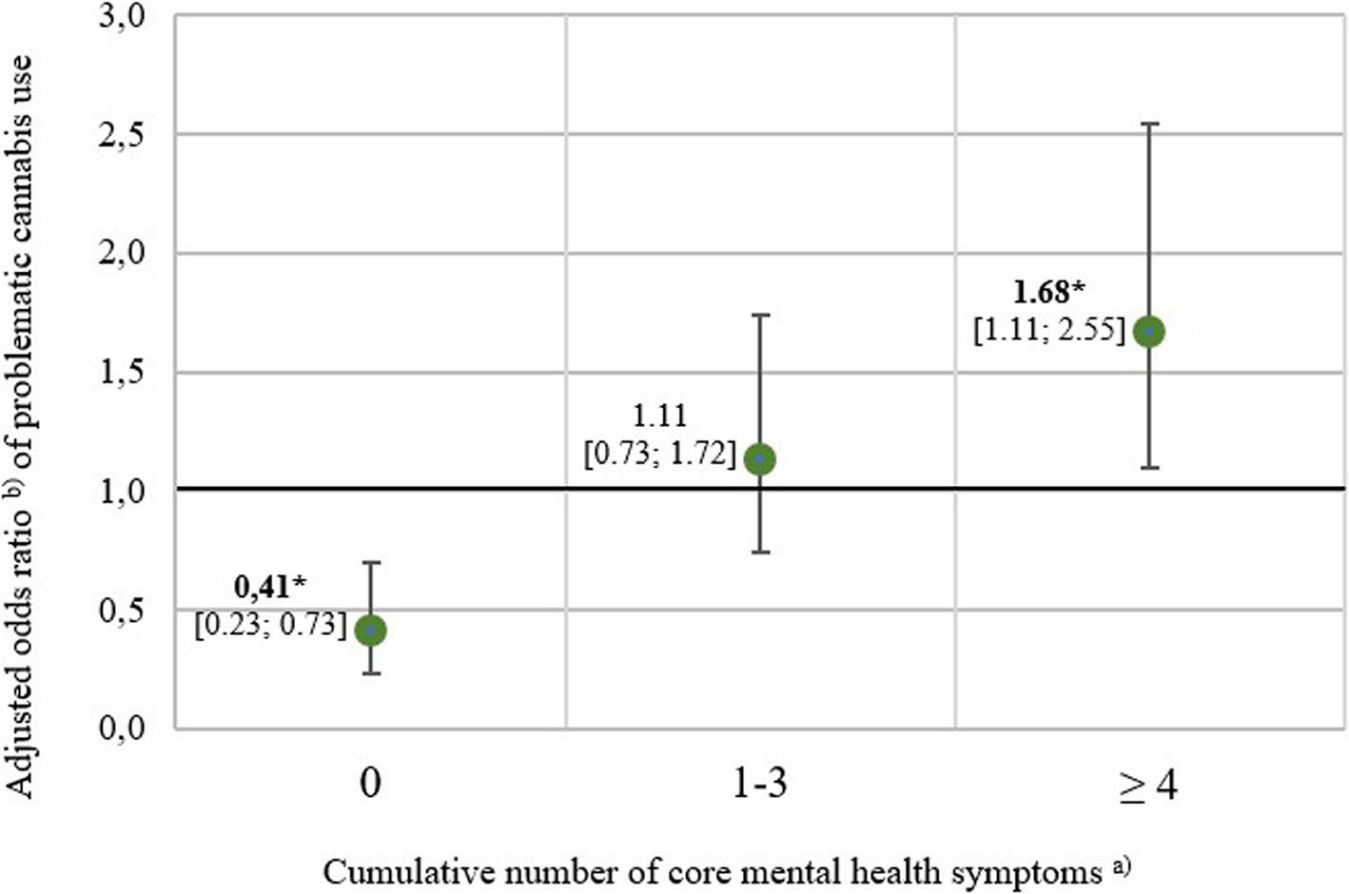
Association between cumulative core mental health symptoms and problematic cannabis use, weighted models (n=938). * Adjusted using the Simes-Benjamini–Hochberg procedure to control the false discovery rate (Simes 1986), significance threshold set at α < 0.05 and indicated with boldface.^a^Cumulative number of core mental health symptoms (range: 0–8), assessed using the DIA-X-SSQ (Wittchen 1997).^b^Each model was adjusted for interview mode, sex, age, education, cannabis consumption frequency, early age of initiation, problematic consumption of amphetamines and current tobacco use

The aORs and corresponding 95% CIs are visualized in Fig. 1. All three models were statistically significant according to the design-based Wald F-test (*p* < 0.001). After controlling for covariates, participants reporting no core mental health symptoms showed a significantly lower chance of problematic cannabis use compared to those with at least one symptom (aOR = 0.41; 95% CI [0.23–0.73]; *p*=0.006). Participants reporting one to three core mental health symptoms did not differ from those without symptoms in their chance of problematic cannabis use (aOR = 1.11; 95% CI [0.72–1.69]; *p*=0.713). In contrast, participants reporting four or more core symptoms had a significantly higher chance of problematic cannabis use compared to those without symptoms (aOR = 1.68; 95% CI [1.12–2.53]; *p*=0.027). Exploratory interaction analyses revealed no statistically significant effect modification by sex (*p*=0.057) or age group (*p*=0.553).

## Discussion

### Summary

Among Germany-residing people who had consumed cannabis in the past-12-month, one in four met the criteria for problematic use. Individuals with problematic use differed from those with non-problematic use across education, consumption patterns, and the problematic use of amphetamines and tobacco. Individuals with problematic cannabis use more often had lower educational attainment, and they also initiated cannabis use at an earlier age more often and reported substantially higher frequencies of use. Almost half of those with problematic use reported consuming cannabis nearly every day. Moreover, they were more likely to engage in current tobacco use and to report problematic amphetamine- and tobacco use. The absence of mental health core symptoms was associated with a reduced chance of problematic cannabis use, whereas the presence of four or more symptoms was associated with a higher chance of problematic cannabis use.

### Covariates of problematic cannabis use

Contrary to previous findings (Hasin et al. 2016; Haug et al. 2017; Rubin-Kahana et al. 2022), the analysis did not show an association between age cohort and problematic cannabis use. As noted in earlier studies, this relationship may be mediated by early age of initiation (Haug et al. 2017). Early cannabis initiation appears to be a stronger predictor than chronological age, likely because it occurs during a period of heightened neurobiological and psychosocial vulnerability, thereby increasing the likelihood of more intensive and dependence-prone use (Crews et al. 2007). This finding stresses the importance of strict enforcement of age restrictions in the access and exposure to cannabis, which are part of the new legislation. Consistent with prior evidence (Fergusson et al. 2003; Lynskey and Hall 2000), the analysis showed that lower educational attainment was also associated with problematic cannabis use. This link may reflect early initiation or social context rather than a direct impact of cannabis on cognition or motivation (Fergusson et al. 2003; Thompson et al. 2019). Nearly half of participants with problematic use consumed cannabis almost daily, compared to fewer than one in ten with non-problematic use. This aligns with prior findings showing that more frequent use increases the likelihood of problematic use (Robinson et al. 2022; Simpson et al. 2021). Individuals with problematic cannabis use were also more likely to currently engage in tobacco use and problematic tobacco use, consistent with earlier research (Agrawal and Lynskey 2009; Hindocha et al. 2021; Weinberger et al. 2020). As previously hypothesized (Agrawal and Lynskey 2009), the shared route of administration - smoking both substances in joints - may reinforce cross-sensitization within the dopaminergic reward system, heightening dependence risk for both substances. This co-use pattern is reportedly more common in Germany than in Canada or the United States (Chu et al. 2023; Hoch 2025; Kotz et al. 2024; Smith et al. 2021), underscoring the need for integrated prevention. Moreover, these findings suggest that tobacco use should be addressed in cannabis cessation programs, and vice versa. Evidence links cannabis use frequency with the use of other substances, as respondents reporting more frequent cannabis use are more likely to engage in high-risk alcohol, amphetamine, and cocaine use (Swift et al. 2012). In our study, persons identified as having problematic use were more likely to report problematic amphetamine use, consistent with findings from young Australians where early and frequent cannabis use predicted later amphetamine initiation (Degenhardt et al. 2007). While international studies show strong comorbidity between problematic cannabis and alcohol use, primarily from the US, Canada (Yurasek et al. 2017), our data did not reveal such an association. This may reflect Germany’s higher and more normalized alcohol consumption (Bloomfield et al. 1995), where risky drinking is common even among individuals without problematic cannabis use. In Germany, 24.4% of past-30-day alcohol consumers report risky use, corresponding to roughly 8.6 million individuals (Olderbak et al. 2024).

### Core mental health symptoms

Regarding mental health burden, individuals with problematic cannabis use exhibited a higher burden of core mental health symptoms. This finding aligns with previous research indicating that individuals with frequent cannabis use and problematic patterns of use, including dependence or CUD, show higher rates of mental health disorders compared to those with non-problematic use (Foster et al. 2018; Lowe et al. 2019; Sorkhou et al. 2024; van der Pol et al. 2013b). Psychosocial and environmental mechanisms interact and contribute to the complex, multifactorial association between problematic cannabis use and mental health disorders (Sagar and Gruber 2025; Urits et al. 2020). Several explanatory theories have been proposed, including the self-medication hypothesis (Khantzian 1997), neurobiological alterations related to cannabinoid exposure (Oleson and Cheer 2012), and shared genetic vulnerability (Kendler et al. 2003). The self-medication hypothesis posits that individuals use cannabis to alleviate symptoms of anxiety, depression, insomnia, or psychological distress (Khantzian 1997). Evidence suggests that a subset of people who report cannabis use describe cannabis consumption as a coping strategy for affective or stress-related symptoms (Lowe et al. 2019; Wallis et al. 2022). Secondly, neurobiological mechanisms emphasize that Δ9-tetrahydrocannabinol (THC) alters neurotransmitter systems, including dopamine, GABA, and glutamate pathways, which are implicated in mood regulation, cognition, and reward processing (Cohen et al. 2019). Cannabis-related perturbations in these systems may precipitate or exacerbate psychotic symptoms, anxiety, and emotional instability (Cohen et al. 2019; Graczyk et al. 2021; Urits et al. 2020). The shared genetic vulnerability hypothesis proposes that familial and genetic risk factors increase susceptibility to both cannabis use and psychiatric outcomes (Kendler et al. 2003). Individuals with pre-existing genetic or familial predispositions for mental disorders may exhibit heightened vulnerability to developing problematic cannabis use (Kendler et al. 2003; Sagar and Gruber 2025). This study extends previous research by examining whether the presence of core symptoms - regardless of diagnostic status - is associated with an increased risk of problematic cannabis use. Individuals without symptoms showed a lower chance of problematic cannabis use, whereas those with four or more core mental health symptoms had nearly twice the chance compared to symptom-free persons who have used cannabis. No associations were observed for those reporting one to three symptoms. Given the potential bidirectional reinforcement between cannabis use and mental health symptoms, where mental health symptoms may increase the likelihood of problematic cannabis use, while cannabis use may exacerbate these symptoms (Radhakrishnan et al. 2023), early identification through low-threshold screening tools based on core mental health symptoms could be an approach to reduce the risk of progression to manifest psychiatric diagnoses and problematic use patterns. Specific treatment approaches should be developed to address the needs of vulnerable populations, particularly individuals with co-occurring mental disorders (Hoch et al. 2025). Most prevention programs in Germany are implemented in school settings (Isensee et al. 2024; Suchert et al. 2024) or in inpatient environments, such as youth welfare facilities (Arnaud 2024). Moreover, treatment programs target individuals who already have manifest mental health diagnoses or exhibit problematic use patterns (Arnaud 2024; Baldus et al. 2011). However, to our knowledge, there are currently no routine screening or prevention approaches specifically designed to identify and support individuals at risk of developing both mental health disorders and problematic cannabis use patterns. Selective and indicated prevention approaches targeting vulnerable populations with elevated risk profiles (Bürger et al. 2020) may represent a promising strategy to address this gap, aiming to prevent the transition from initial cannabis use to problematic patterns of use. For primary prevention, the observed association between problematic cannabis use and mental health symptoms underscores the relevance of life skills training, which aims to enhance overall personal resilience.

Lastly, partial legalization in Germany may increase the availability of cannabis and drive down prices, potentially facilitating access and use (Hall and Lynskey 2020; Manthey et al. 2023). Cannabis use frequency and the progression to problematic use might increase more rapidly following legalization among individuals with mental health problems compared to those without such vulnerabilities (Hyatt et al. 2025). Thus, increased availability and easier access may contribute to a faster transition from non-problematic to problematic cannabis use, particularly among individuals with pre-existing mental health conditions. However, the extent of such effects remains uncertain and likely depends on regulatory frameworks, market dynamics, and individual vulnerability factors.

### Strengths and limitations

The results presented are subject to the following design-inherent limitations. First, the DIA-X-SSQ is a brief screening tool with limited specificity - ranging from 12% for somatoform disorders to 49% for depression. However, it shows adequate sensitivity: if a symptom is denied, the likelihood of the respective disorder remains low (about 1% for anxiety disorders to 4% for substance use disorders) (Wittchen 1997). Moreover, the severity of individual symptoms was not assessed, precluding conclusions about the associations of specific items. The assessment of cannabis use, related consumption patterns, and core mental health symptoms was based on self-reports and is therefore susceptible to biases such as social desirability and recall bias. A small number of cases had to be excluded from the analyses due to missing data. While the extent of these exclusions was limited, a potential influence on the results cannot be entirely ruled out, although a systematic bias appears unlikely. Important risk factors associated with problematic use, such as biological predispositions or THC potency (Connor et al. 2021), were not accounted for. Finally, due to the cross-sectional design, causality cannot be established.

To our knowledge, this is the first study to investigate the association between core mental health symptoms and problematic cannabis use in a nationally representative sample while accounting for multiple covariates. Most research comparing individuals with and without problematic cannabis use has been conducted in the United States, where consumption patterns differ from those in Europe (Cousijn et al. 2024). With the legalization of cannabis in Germany, identifying both risk and protective factors is essential for assessing potential health impacts and informing public health strategies. Baseline data collected prior to legalization are essential as a reference point for comparisons between the pre- and post-legalization phases. Future analyses will examine whether risk factors for problematic cannabis use have changed following legalization and, if so, to what extent. Our findings add value by providing nationally representative data on the differences between individuals with problematic cannabis use patterns and those with non-problematic use patterns in Germany. However, representativeness is limited due to selective nonresponse, resulting in a response rate of 35%. To minimize potential bias from nonresponse, post-stratification weights were applied to align the marginal distributions of key external characteristics with those of the target population.

## Conclusion

In light of cannabis legalization in Germany, identifying risk factors for problematic use remains essential. This study contributes to a more comprehensive understanding of problematic cannabis use in Germany by delineating differences between individuals with and without problematic use and examining their association with core mental health symptoms. The findings suggest that such symptoms, even in the absence of a clinical diagnosis, may be linked to problematic use. These results highlight the importance of early intervention strategies, such as brief screening instruments, to identify individuals at risk.

## Supplementary Information

Supplementary Material 1

## Data Availability

The data analyzed for this study are publicly accessible via GESIS ([**www.gesis.org**] (**http:/www.gesis.org**)).
