# Supplementary material for: Who is at risk? Core mental health symptoms and problematic cannabis use in Germany
Source: J Cannabis Res. 2026 Jun 5;8:71. doi: 10.1186/s42238-026-00430-y (PMC13237940; doi:10.1186/s42238-026-00430-y)
Supplement: Supplementary file 1 — Supplementary Material 1 [file 42238_2026_430_MOESM1_ESM.docx]

**SUPPLEMENTARY MATERIAL**

**Supplementary material 1**. Mental health core symptoms assessed with the DIA-X-Core Screening Questionnaire (20)

| *Core symptom* |  | *German* | *English Translation* |
| --- | --- | --- | --- |
| Agoraphobia |  | Litten Sie unter unbegründet starken Ängsten vor öffentlichen Plätzen, Verkehrsmittel zu benutzen oder in Geschäfte zu gehen? | Did you suffer from excessively strong and unfounded fears of public places, using public transportation, or going into shops? |
| Depression |  | a) Litten Sie über mehr als zwei Wochen fast täglich unter Traurigkeit oder Niedergeschlagenheit?  b) Litten Sie über mehr als zwei Wochen fast täglich unter Interessenverlust, Müdigkeit oder Energielosigkeit? | a) Did you suffer from sadness or feeling down nearly every day for more than two weeks?  b) Did you suffer from loss of interest, fatigue, or lack of energy nearly every day for more than two weeks? |
| Generalized anxiety disorder |  | Gab es eine Zeitspanne von einem Monat oder länger, in der Sie sich häufig Sorgen gemacht haben und sich ängstlich, angespannt oder voller ängstlicher Besorgnis gefühlt haben? | Was there a period of one month or longer during which you frequently felt worried, anxious, tense, or filled with anxious concerns? |
| Mania |  | Waren Sie über mehrere Tage hinweg ungewöhnlich glücklich, überdreht oder reizbar, so dass sich Freunde oder Angehörige Sorgen machten? | Were you unusually happy, overly excited, or irritable for several days to the extent that friends or family became concerned? |
| Panic disorder |  | Hatten Sie einen Angstanfall – manche nennen das auch Panikattacke oder Angstattacke – bei dem Sie ganz plötzlich von einem Gefühl starker Angst, Beklommenheit oder Unruhe überfallen wurden? | Have you ever had an anxiety attack – sometimes called a panic attack – where you were suddenly overwhelmed by a feeling of intense fear, distress, or nervousness? |
| Post-traumatic-stress-disorder |  | Gab es in Ihrem Leben extrem belastende schreckliche Ereignisse oder Katastrophen, die Sie in den letzten 12 Monaten noch beschäftigten? | Have there been extremely distressing, terrible events, or disasters in your life that have continued to trouble you in the past 12 months? |
| Social phobia |  | Litten Sie unter unbegründet starken Ängsten in sozialen Situationen, wie mit Anderen zu reden, etwas in Gegenwart Anderer zu tun oder im Mittelpunkt der Aufmerksamkeit Anderer zu stehen? | Did you suffer from excessively strong unfounded fears in social situations, such as speaking with others, doing something in front of others, or being the center of attention? |
| Somatoform disorder |  | Litten Sie mehrere Monate lang unter körperlichen Beschwerden oder Schmerzen, für die Ihr Arzt keine eindeutige Erklärung finden konnte? | Did you suffer for several months from physical complaints or pain for which your doctor could not find a clear explanation? |

***Note.*** Translation of the original items conducted using *DeepL* (DeepL SE, Cologne, Germany).
